# BAFF induces CXCR5 expression during B cell differentiation in bone marrow

**DOI:** 10.1016/j.bbrep.2023.101451

**Published:** 2023-03-07

**Authors:** Hajime Koizumi, Wataru Fujii, Chizu Sanjoba, Yasuyuki Goto

**Affiliations:** a Laboratory of Molecular Immunology, Department of Animal Resource Sciences, Graduate School of Agricultural and Life Sciences, The University of Tokyo, Japan; b Laboratory of Biomedical Science, Department of Veterinary Medical Sciences, Graduate School of Agricultural and Life Sciences, The University of Tokyo, Japan

**Keywords:** BAFF, Bone marrow, B cell development, CXCR5

## Abstract

B cell activating factor (BAFF) plays an important role in antibody production through differentiation and maturation of B cells mainly in secondary lymphoid organs. On the other hand, the role of BAFF in the bone marrow, the primary lymphoid organ of B cell development, has not been well elucidated. Here, effects of BAFF in bone marrow B cell development were examined by using BAFF-deficient mice. When mRNA expression levels of B cell differentiation markers including *Cd19*, *Bcl2*, *Igμ*, *Il7r* and *Cxcr5* were compared between bone marrow of wild-type and BAFF-KO mice, a lower level of *Cxcr5* expression was found in the KO mice. Additionally, protein expression of CXCR5 on IgM^+^ cells in the bone marrow was decreased by BAFF deficiency. In vitro studies also confirmed the effect of BAFF on CXCR5 by IgM^+^ cells; culturing bone marrow cells from BAFF-KO mice with BAFF in vitro increased the proportion of CXCR5^+^ cells in IgM^+^ cells compared with non-treated bone marrow cells. In addition, BAFF synergized with TNF-α and IL-6 to increase the expression of CXCR5^+^ on IgM^+^ cells. The BAFF-mediated up-regulation of CXCR5 expression was reproduced by using CD19^+^ cells purified from BAFF-KO bone marrow cells, suggesting that BAFF directly affects B-lineage cells in bone marrow to promote CXCR5 expression. Together, this study suggests that BAFF has an important role in B cell differentiation in bone marrow by directly inducing CXCR5 expression which affect their migration to secondary lymphoid organs.

## Highlights

• BAFF plays an important role in differentiation and maturation of B cells in secondary lymphoid organs.
• However, the role of BAFF in B cell development in bone marrow remained elusive.
• We found that BAFF induces CXCR5 expression on IgM ^+^ cells in bone marrow.
• BAFF seems to have important roles in B cell development not only in secondary lymphoid organs but also in bone marrow.

## 1 Introduction

Differentiation and maturation of B cells into plasma cells involves both primary lymphoid organs, mainly bone marrow, and secondary lymphoid organs such as spleen and lymph nodes. In the bone marrow, lymphoid progenitors differentiate into pro-B cells, pre-B cells, and immature B cells in that order [1,2]. Pre-B cells expressing pre-B receptor that has a certain affinity to antigen differentiate into immature B cells that express IgM on their cell surface as B cell receptors (BCR) [3]. The surviving immature B cells flow out into blood vessels, circulate and migrate to secondary lymphoid tissues including the spleen [4]. Immature B cells allowed to migrate to primary follicles differentiate into naive B cells [5]. Then, upon antigen experience the B cells terminally differentiate into plasma cells producing antibodies. Together, antibody production by plasma cells should be affected if any step of the B cell differentiation is hampered.

One of the cytokines involved in B cell differentiation and maturation is B-cell activating factor (BAFF). BAFF is a cytokine belonging to the tumor necrosis factor superfamily secreted mainly by monocytes, macrophages, T cells and dendritic cells [6,7]. BAFF is a cytokine that regulates B cell survival, differentiation, and maturation through its three receptors, BAFF receptor (BAFF-R), B cell maturation antigen (BCMA), and transmembrane activator and calcium-modulator and cyclophilin ligand interactor (TACI), which are mainly expressed on B cells in secondary lymphoid organs [8]. BAFF/BAFF-R signaling contributes to the survival of naive B cells and antigen-presenting germinal center B cells by inducing the expression of the anti-apoptotic molecule B cell lymphoma 2 (BCL2) [[9], [10], [11]]. BAFF/BCMA signaling similarly inhibits plasma cell apoptosis by inducing expression of myeloid cell leukemia sequence 1, which is one of the BCL2 family, contributing to the long-term survival of plasma cells [12]. BAFF signaling through TACI induces a class-switch of immunoglobulins in antigen-presenting B cells, contributing to the production of antibodies such as IgG, IgE and IgA [13]. In particular, class switch of antibodies to thymus-independent (TI) antigens such as bacterial capsular polysaccharides [14], which do not require supplemental stimulation from helper T cells, require stimulation by BAFF [15]. Thus, BAFF contributes to the differentiation of immature B cells into plasma cells mainly in secondary lymphoid organs. It is known that BAFF-KO mice show a significant decrease in the production of immunoglobulins even without infection or exposure to antigens (here we define such status as ‘steady-state’) or antigen-specific antibodies upon antigen stimulation. For example, in experiments where BAFF-KO mice were immunized with nitrophenyl acetyl-keyhole limpet hemocyanin, a TD antigen, or trinitrophenyl-Ficoll, a TI-2 antigen, there was a marked decrease in antigen-specific IgG production to either antigen [16]. Based on the roles of BAFF in survival, differentiation, and antigen-specific activation described above, as well as the marked decrease in antigen-specific antibody production in BAFF-KO mice, BAFF has been considered as an essential factor in B cell maturation and antibody production.

Although roles of BAFF in antibody production have been studied mostly from the point of view of B cell activation/maintenance in secondary lymphoid tissues, to fully understand the role of BAFF in B cell maturation it is necessary to investigate the function of BAFF before antigen experience. One study has shown that the number of immature B cells produced in the bone marrow of BAFF-KO mice is comparable to that of WT mice, whereas the number of CD19^+^ B cells in the spleen is greatly reduced [16]. Therefore, it has been considered that BAFF affects humoral response by augmenting the settlement of B cells into primary follicles of the spleen but not influencing the quality and quantity of immature B cells produced in the bone marrow [16]. In contrast, there are some studies implying that BAFF contributes to the bone marrow IgM^+^ B cell differentiation at steady-state. For example, half of the immature B cells in the bone marrow express BAFF-R [17], and stimulation of immature B cells with BAFF in vitro leads to differentiation into naive B cells and increased survival time [18,19]. In addition, while autoreactive immature B cells have low BAFF-R expression, immature B cell with high BCR signaling has high BAFF-R expression, suggesting that BAFF contributes to the elimination of autoreactive B cells [18].

Reduction of B cells in the spleen by BAFF deficiency [16] may be because of altered C-X-C motif chemokine receptor 5 (CXCR5) expression by immature B cells in bone marrow. CXCR5 serves as a receptor for C-X-C motif chemokine ligand 13 (CXCL13) and both are needed for B-cell homing to follicles in lymph nodes as well as in spleen [20]. Chemoattraction by CXCR5 expressed by IgM^+^ B cells and CXCL13 expressed by follicular stromal cells are necessary for IgM^+^ B cells to migrate to primary follicles in the spleen [21]. IgM^+^ B cells with strong autoreactivity, which are not eliminated in bone marrow, have weaker expression of CXCR5, making them difficult to settle in the primary follicle of the spleen [22]. Additionally, B cell regions and germinal centers in the spleen are not formed in CXCR5-KO mice after antigen presentation [21]. In CXCR5-KO mice, the formation of germinal center-like micro-spaces near the periphery of the T-cell region results in a limited effect on antibody production [23], but antigen-specific antibody production is greatly reduced during viral infection [24]. It has been reported that BAFF-KO mice and BAFF-R mutant mice fail to maintain germinal center formation after antigen presentation and secondary lymphoid follicles do not mature [25]. Although decrease in the percentage of B cells that migrate from the bone marrow to the spleen at steady-state BAFF-R mutant mice indicates that B cell migration is altered [26], it has not been reported that BAFF is involved in migration ability through chemokines. Here we investigated whether BAFF affects CXCR5 expression on immature B cells in the bone marrow at steady-state.

## 2 Materials and methods

### 2.1 Mice

Female BALB/cA mice were purchased from CLEA Japan, Tokyo, Japan. BAFF-knock out (BAFF-KO) mice on a BALB/cA background were produced by CRISPR/Cas offset-nicking method [27]. All mice were maintained under specific-pathogen-free conditions in a level 2 physical containment facility. All animal experiments were reviewed and approved by an institutional animal committee at the Graduate School of Agricultural and Life Sciences, The University of Tokyo (Approval No. P19–002). This study complies with ARRIVE guidelines, and was carried out in accordance with the National Institutes of Health guide for the care and use of Laboratory animals (NIH Publications No. 8023, revised 1978).

### 2.2 Harvest of the bone marrow and spleen cells from mice

The Bone marrow cells from wild-type (WT) and BAFF-KO mice were collected by flushing femurs and tibias with phosphate-buffered saline (PBS). Single cell suspension of the bone marrow and spleen cells were prepared by passing through a 70 μm cell strainer (BD Pharmingen, CA, USA). Erythrocytes in each preparation were lysed with Red Blood Cell Lysing buffer (Sigma Aldrich, MO, USA), and the remaining cells were washed three times with PBS.

### 2.3 Quantitative reverse Transcription-PCR

RNA from the spleen and bone marrow was extracted using TRIzol (Thermo Fisher Scientific, MA, USA) and cDNA was synthesized from the RNA by reverse transcription. Only spleen tissues were homogenized with 1 ml TRIzol and φ1.0 glass beads (Tomy Seiko, Tokyo, Japan) in the 2 ml tube using Micro Smash MS100R (Tomy Seiko) at 4 °C for 3 min. After transfer to a new 1.5 ml tube, the supernatants were mixed with 200 μl of chloroform and centrifuged at 12,000×*g* for 15 min at 4 °C. The aqueous phase were recovered and mixed with 0.5 ml 2-propanol (Fujifilm Wako, Osaka, Japan) and centrifuged at 12,000×*g* for 10 min at 4 °C After washing with 1 ml of 75% ethanol, RNA was dissolved in UltraPure distilled water (Thermo Fisher Scientific). The concentration of total RNA was measured by DU 730 Life Science UV/vis spectrophotometer (Beckman Coulter, CA, USA) and 2 μg of total RNA was used as the template for the synthesis of 20 μl cDNA. The mixture including 1.25 μM oligo (dT)16, and 0.15 mM dNTPs (Thermo Fisher Scientific) with template RNA in the tube was incubated for 5 min at 65 °C. After adding 5 × first strand buffer and 10 mM DTT (Thermo Fisher Scientific), 200 U M-MLV (Thermo Fisher Scientific) was added and the tube was incubated for 50 min at 37 °C and 15 min at 70 °C.

Real-time PCR assay was carried out using 0.5 μl of cDNA as a template, 10 μl of SYBR Select Master Mix (Thermo Fisher Scientific) and 0.25 mM primers listed in Table 1 on the ABI Prism 7000 Sequence Detection System (Thermo Fisher Scientific). Data were analyzed by 2^−ΔΔCt^ methods and normalized by GAPDH. The thermal cycling conditions for the PCR were 50 °C for 2 min followed by 95 °C for 10 min and 40 cycles of 95 °C for 15 s and 60 °C for 1 min.

### 2.4 Cell culture and cytokine stimulation assays

1.0 × 10^6^ bone marrow cells from BAFF-KO mice in complete DMEM, i.e., DMEM supplemented with 10% heat-inactivated fetal bovine serum (Sigma Aldrich), 100 U/ml penicillin and 100 μg/ml streptomycin (Fujifilm Wako), were plated in a 96-well round-bottom plate (Thermo Fisher Scientific) and then stimulated with 100 ng/ml of recombinant murine BAFF (rmBAFF) (R&D SYSTEMS #8876-BF-010/CF), 100 ng/ml of rmTNF-α (R&D SYSTEMS #410-MT-010), 50 ng/ml of rmIL-6 (R&D SYSTEMS #406-ML-005), or medium alone. After 48 h of cultivation in 5% CO_2_ at 37 °C, the plate was centrifuged at 300×*g* for 5 min. The culture supernatant was removed, and cells were washed two times by PBS with 1% HI-FBS for flow cytometry analysis preparation.

### 2.5 CD19^+^ cell separation from the bone marrow cells

5 μl of anti-CD19 microbeads (Miltenyi Biotec, NRW, Germany) were added to 1.0 × 10^7^ bone marrow cell suspension from BAFF-KO mice in MACS buffer (PBS with 0.5% HI-FBS and 2 mM of EDTA). The cell suspension after 15 min of cultivation at 4 °C was centrifuged at 300×*g* for 5 min, and washed with MACS buffer and kept at 4 °C. Separation was performed by using MiniMACS™ separator (Miltenyi Biotec) and MS column (Miltenyi Biotec). Before cell separation, MS column was placed on the separator, and rinsed with 500 μl of the buffer. Cells were applied onto the rinsed MS column, and washed 3 times with the buffer, and then flow-through was collected as CD19^−^ cell population. After the column was removed from the separator, 1 ml of buffer were added, and then the fraction was collected with the buffer as CD19^+^ cell population. CD19^+^ cells were isolated with >95% purity.

### 2.6 Flow cytometric analysis of the bone marrow cells and splenocytes

Single cell suspensions of the bone marrow and spleen cells in PBS with 1% HI-FBS were incubated with FITC-conjugated *anti*-IgM monoclonal antibody and PE-conjugated *anti*-CXCR5 monoclonal antibody (BD Pharmingen) for 1 h on ice and washed three times with PBS plus 1% HI-FBS. After washing, the cells were fixed by BD Cytofix/Cytoperm Fixation/Permeabilization solution (BD Pharmingen), and then washed again. At least 100,000 cells per sample were analyzed on the BD FACSVerse™, and data analysis was performed using BD FACSuite™ software. The lymphocytes were gated in the FSC-SSC dot plots, and the number of lymphocyte-gated IgM^+^ cells and CXCR5^+^ cells were analyzed by quadrant analysis of 2-dimensional dot plots.

### 2.7 Statistical analyses

Differences among groups were analyzed by either unpaired *t-*test, one-way ANOVA followed by Bonferroni multiple comparisons test or two-way ANOVA followed by Bonferroni multiple comparisons test with GraphPad Prism 9 software (GraphPad Software, Inc., CA, USA). *P* values less than 0.05 were considered significantly different.

## 3 Results

### 3.1 Decreased *Cxcr5* gene expression in the bone marrow cells of BAFF-KO mice

**Fig. 1:**
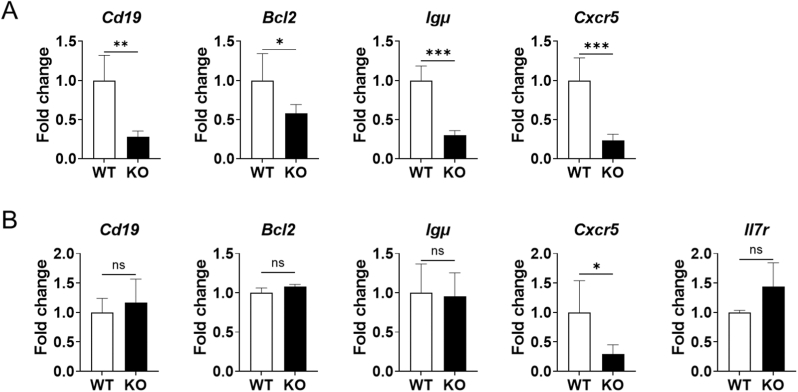
Suppressed *Cxcr5* expression in bone marrow cells of BAFF-KO mice (A) The gene expression of *Cd19, Bcl2, Igμ* and *Cxcr5* in the spleen of WT and BAFF-KO mice analyzed by qPCR. (B) The expression of *Cd19, Bcl2, Igμ, Il7r* and *Cxcr5* in the bone marrow of WT and BAFF-KO mice analyzed by qPCR. Graphs show mean and SD of each group (n = 5). *P < 0.05; **P < 0.01; ***P < 0.001; ns, not significant by unpaired *t*-test.

Previous research showed the remarkedly decreased number of CD19^+^ cells at steady-state in BAFF-KO mice spleen [27]. Thus, expression of *Cd19, Bcl2, Igμ*, and *Cxcr5* by spleen cells were evaluated. Expression of all the genes were significantly reduced in the spleen of BAFF-KO mice compared with WT mice (Fig. 1A). Previous research showed that B cell development has been remarkedly suppressed in BAFF-KO mice spleen, not in the bone marrow [16]. To re-evaluate the role of BAFF in B cell differentiation in the bone marrow, qPCR was performed on cDNA from the bone marrow of WT and BAFF-KO mice using several marker genes that are expressed specifically in the bone marrow B cells. The qPCR results showed no difference in the expression levels of *Il7r, Bcl2, Cd19* and *Igμ* in the bone marrow cells between WT and BAFF-KO mice (Fig. 1B). On the other hand, *Cxcr5* gene expression in the bone marrow cells of BAFF-KO mice was significantly lower than WT mice. (Fig. 1B).

### 3.2 Decreased CXCR5 protein expression on IgM ^+^ cells in the bone marrow and spleen of BAFF-KO mice

**Fig. 2:**
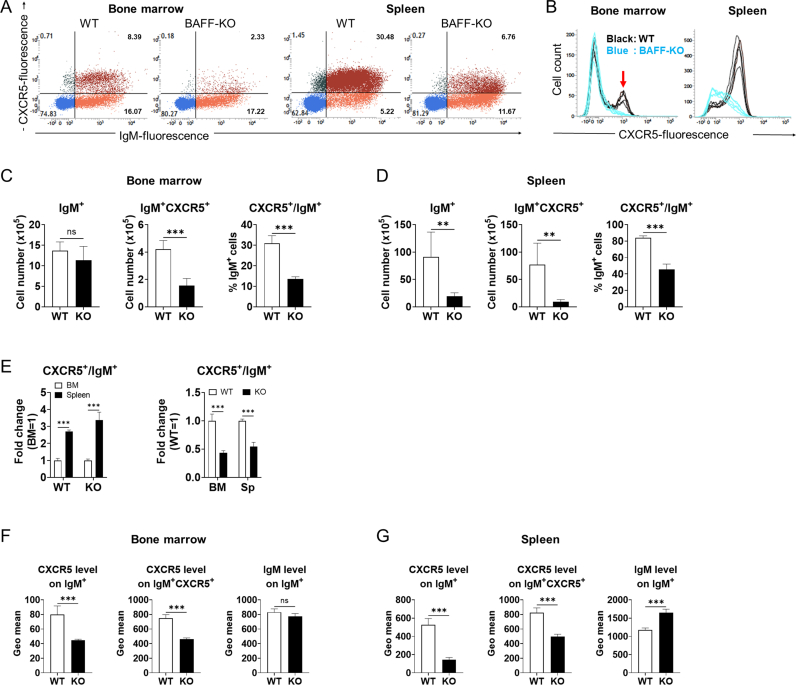
Decreased CXCR5 expression of IgM ^+^ cell in bone marrow of BAFF-KO mice Bone marrow and spleen cells from WT and BAFF-KO mice were collected and stained with FITC-αIgM and PE-αCXCR5. (A) Representative profiles of flow cytometry for lymphocyte-gated bone marrow and spleen cells. The numbers in the graphs show the proportion of cells in each quadrant. (B) CXCR5 fluorescence intensity of IgM^+^ cells from bone marrow and spleen (Black, WT; Blue, BAFF-KO). CXCR5^high^ IgM ^+^ bone marrow cells, which were detectable only in WT mice, are pointed by an arrow. (C, D) Total cell numbers of IgM^+^ cells and IgM^+^CXCR5^+^ cells as well as the proportion of CXCR5^+^ cells in IgM^+^ cells in bone marrow (C) and spleen (D) for both WT and BAFF-KO mice. (E) The proportion of CXCR5-expressing cells in IgM^+^ cells of the bone marrow and spleen from WT and BAFF-KO mice represented as relative fold change to bone marrow or relative fold change to WT mice. (F, G) Geometric mean of CXCR5 fluorescence on IgM^+^ cells and IgM^+^CXCR5^+^ cells, and IgM fluorescence on IgM^+^ cells of the bone marrow (F) and spleen (G) in WT and BAFF-KO mice. Graphs show mean and SD of each group (n = 5). **P < 0.01; ***P < 0.001; ns, not significant by either unpaired *t*-test or two-way ANOVA followed by Bonferroni multiple comparisons test. (For interpretation of the references to colour in this figure legend, the reader is referred to the Web version of this article.)

CXCR5 is a chemokine receptor expressed on IgM^+^ cells and is involved in migration to secondary lymphoid tissues [21]. Therefore, CXCR5 protein expression of IgM^+^ cells in the bone marrow and spleen of WT and BAFF-KO mice were examined by flow cytometry. IgM^+^ CXCR5^+^ bone marrow cells in BAFF-KO mice were reduced to less than 50% compared with that of WT mice (Fig. 2A–D). On the other hand, there was no difference in the number of IgM^+^ cells in bone marrow between BAFF-KO and WT mice (Fig. 2C, left). The ratio of CXCR5^+^ cells in IgM^+^ bone marrow cells of BAFF-KO mice was less than 15%, whereas that of WT mice was ∼30% (Fig. 2C, right). In the spleen, the total cell number was remarkedly decreased in BAFF-KO mice as previously reported [27]. The number of IgM^+^ and IgM^+^ CXCR5^+^ splenocytes of BAFF-KO mice were both significantly decreased to about 30% and 12% compared to that of WT mice, respectively (Fig. 2D: left, middle). The ratio of CXCR5^+^ cells in IgM^+^ cells in WT and BAFF-KO mouse spleen was about 80% and 50%, respectively (Fig. 2D: right). Despite the decrease in CXCR5 expression by IgM^+^ bone marrow cells in BAFF-KO mice, the molecule seemed still responsible for migration of IgM^+^ cells to the spleen even in the KO mice; in both WT and KO mice the ratio of CXCR5^+^ cells to IgM^+^ cells in the spleen was about three times as much as that of the bone marrow, and the lower proportion of CXCR5^+^ cells in IgM^+^ cells in the spleen of BAFF-KO mice was reflective of that in the bone marrow (Fig. 2E). CXCR5 expressions on IgM^+^ cells and IgM^+^ CXCR5^+^ cells of the bone marrow and spleen were both significantly decreased in BAFF-KO mice compared to WT mice (Fig. 2F and G: left, middle). On the other hand, IgM expression of IgM^+^ cells in the bone marrow did not differ between WT and BAFF-KO mice, whereas that in BAFF-KO mouse spleen was increased compared to that in WT mouse spleen (Fig. 2F and G: right).

### 3.3 Direct induction of CXCR5 expression in IgM ^+^ bone marrow cells from BAFF-KO mice by BAFF

**Fig. 3:**
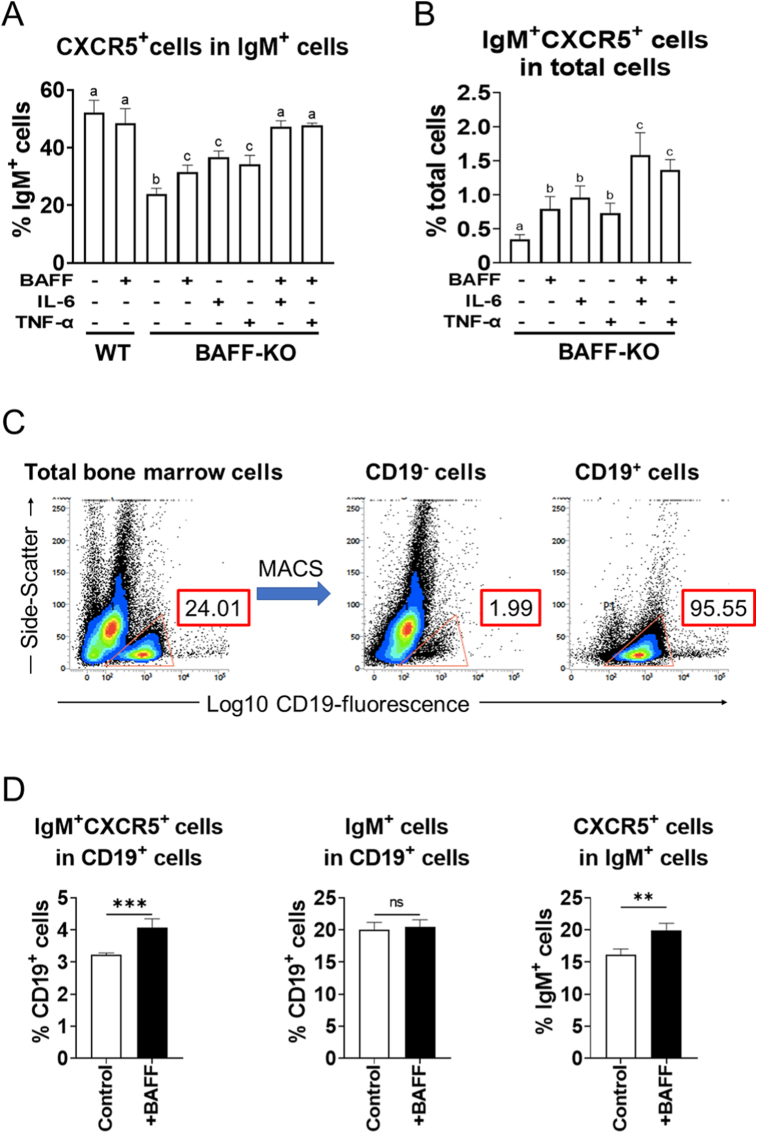
Direct induction of CXCR5 expression in IgM ^+^ bone marrow cells from BAFF-KO mice by BAFF (A, B) Bone marrow cells from WT and BAFF-KO mice were cultured for 48 h in the presence of rmBAFF, rmIL-6, and/or rmTNF-α. Bone marrow cells were co-stained with FITC-αIgM and PE-αCXCR5, and lymphocyte-gated cells were analyzed by flow cytometry. (A) The proportion of CXCR5^+^ cells in IgM^+^ cell. (B) The proportion of IgM^+^CXCR5^+^ cells in lymphocyte-gated cells. Graphs show mean and SD of each group (n = 5). Different letters on the bars in the graphs indicate statistical difference between groups. (C, D) Bone marrow cells from BAFF-KO mice were separated to CD19^−^ and CD19^+^ cell populations by MACS. CD19^+^ cells were cultured with rmBAFF for 48 h. Cells were co-stained with FITC-αIgM and PE-αCXCR5, and lymphocyte-gated cells were analyzed by flow cytometry. (C) Representative profiles of flow cytometry for total, CD19^−^, and CD19^+^ bone marrow cells. The numbers in the graphs show the proportion of cells in the gated areas. (D) The proportions of IgM^+^ cells and IgM^+^CXCR5^+^ cells in lymphocyte-gated CD19^+^ cells as well as the proportion of CXCR5^+^ cells in IgM^+^ cells. Graphs show mean and SD of each group (n = 5). **P < 0.01; ***P < 0.001; ns, not significant by unpaired *t*-test.

To confirm whether BAFF induces CXCR5 expression in IgM^+^ cells, WT and BAFF-KO mouse bone marrow cells were cultured with and without rmBAFF for 48 h, and analysis on the number of IgM- and CXCR5-expressing cells were performed by flow cytometry. Since IL-6 and TNF-α have been reported to be cytokines that induce the expression of CXCR5 [28,29], cells stimulated by rmIL-6 or rmTNF-α alone, or co-stimulated by rmIL-6 + rmBAFF or rmTNF-α + rmBAFF were also prepared for flow cytometry analyses. The proportion of CXCR5^+^ cells in IgM^+^ bone marrow cells of BAFF-KO mice was increased by BAFF stimulation, whereas that of WT was unchanged (Fig. 3A). Furthermore, the ratio of CXCR5^+^ cells in IgM^+^ bone marrow cells of BAFF-KO mice was increased by incubation with rmIL-6 and rmTNF-α, and the increase was intensified by BAFF co-stimulation (Fig. 3A). rmTNF-α, rmIL-6, and rmBAFF induced CXCR5 expression in the bone marrow IgM^+^ cells from BAFF-KO mice to the same extent. In addition, co-stimulation with rmBAFF + rmIL-6 or rmBAFF + rmTNF-α restored the expression of CXCR5 in IgM^+^ cells in the bone marrow of BAFF-KO mice to the extent in that of WT (Fig. 3A). The proportion of IgM^+^ CXCR5^+^ cells in total cells was significantly increased by rmBAFF, rmIL-6, or rmTNF-α stimulation alone, and was significantly increased by co-stimulation (Fig. 3B).

To confirm whether BAFF stimulation directly induces CXCR5 expression on IgM^+^ bone marrow cells, CD19^+^ cells isolated by MACS from BAFF-KO mouse bone marrow were stimulated with rmBAFF and analyzed on the number of IgM^+^ and CXCR5^+^ cells by flow cytometry. CD19^+^ cells were isolated with >95% purity by MACS (Fig. 3C). The proportion of IgM^+^ CXCR5^+^ cells in total CD19^+^ bone marrow cells stimulated by rmBAFF were significantly increased compared to control, whereas the proportion of IgM^+^ cells in CD19^+^ bone marrow cells did not differ between the control and rmBAFF-stimulated group (Fig. 3D). The proportion of IgM^+^ CXCR5^+^ cells in IgM^+^ cells were also increased by BAFF stimulation (Fig. 3D).

## 4 Discussion

BAFF is known to affect B cell differentiation and maturation, mainly in secondary lymphoid tissues [30]. On the other hand, the role of BAFF in the bone marrow has been little studied, especially in relation to chemokine receptor expression on B cells which can also affect B cell migration to secondary lymphoid tissues [21] and antibody production [24,31]. BAFF-KO and CXCR5-KO mice show similar phenotypes, such as the disappearance of B-cell regions [16,21] in the spleen at steady-state and impaired germinal center formation [21,25] after antigen stimulation. Since the migration of B cells in the spleen of BAFF-KO mice is already altered at steady-state [27], and CXCR5 is expressed on the IgM^+^ B cell stage in the bone marrow [28], it is conceivable that BAFF is involved in CXCR5 expression during B cell development in the bone marrow. Therefore, the purpose of this study was to clarify whether BAFF affects CXCR5 expression in IgM^+^ cells in the bone marrow.

A previous study using BAFF-KO mice has shown that CD19^+^ cells in the spleen are significantly reduced [27]. Therefore, we decided to check the gene expression levels of *Cd19, Bcl2, and Igμ,* which are expressed during B cell differentiation in the spleen and bone marrow [[32], [33], [34]]. The marked decrease in expressions of *Cd19, Igμ, Bcl-2* (Fig. 1A) suggests decreased B cell numbers in the spleen by BAFF deficiency as previously reported [27]. CXCR5 is expressed on both T cells and B cells in the spleen [35] and BAFF deficiency leads to decrease in the number of T cells in spleen as well [27]. However, it is known that CXCR5 expression on T cells is upregulated after antigen presentation to strengthen the connection with B cells [35]. Therefore, it is considered that the decreased *Cxcr5* gene expression in the spleen of BAFF-KO mice is mainly due to the decrease in B cells. In bone marrow, we found no changes in expressions of *Cd19, Il7r*, and *Igμ* by BAFF deficiency. IL-7R is highly expressed between pro-B cell and pre-B cell stages in the bone marrow [36]. Together, it is suggested that B cell differentiation in the bone marrow is not stagnated by BAFF deficiency [16]. On the other hand, *Cxcr5* expression was significantly reduced by BAFF deficiency (Fig. 1B). In the bone marrow, CXCR5 is mainly expressed after IgM^+^ expression in B cells [28], indicating that CXCR5 protein expression on IgM^+^ cells is declined.

In bone marrow, the population of IgM^+^ cells highly expressing CXCR5 seems to decrease by BAFF deficiency (Fig. 2A–C). IgM^+^ cells in bone marrow include immature B cells (IgM^+^IgD^−^) and circulating mature B cells (IgM^low^IgD^high^) [16]. BAFF deficiency did not affect the number of IgM^+^ cells and their IgM expression in the bone marrow (Fig. 2C, F), suggesting that the decreased CXCR5 expression is not biased toward either immature or mature B cells. On the other hand, IgM expression levels of IgM^+^ cells in the spleen were increased by BAFF deficiency (Fig. 2G). IgM expression of B cells decreases from immature B cell to mature B cell stage [37] and BAFF signaling is essential for the survival of mature B cells [38]. In this study, IgM^+^ cells in the spleen decreased by BAFF deficiency (Fig. 2D). Thus, BAFF deficiency may stall the differentiation into mature B cells in the spleen as previously reported [18].

The effects of BAFF in CXCR5 expression on B-lineage cells in bone marrow were also confirmed by in vitro experiments cultivating bone marrow cells from BAFF-KO mice with BAFF. In the experiments, BAFF showed equivalent activity of CXCR5 up-regulation to IL-6 and TNF-α, both of which are known to induce CXCR5 expression on B-lineage cells [28,29]. Since increase in CXCR5 expression in IgM^+^ cells induced by IL-6 or TNF-α was enhanced by BAFF co-stimulation (Fig. 3A and B), BAFF may induce CXCR5 expression on the bone marrow B cells by a different pathway with TNF-α or IL-6. Also, BAFF seems to act directly on B-lineage cells for differentiation in bone marrow (Fig. 3D). BAFF/BAFF-R signaling promotes the expression of anti-apoptotic molecules in B cells via the noncanonical NF-kB pathway [39]. The activation through the noncanonical NF-kB pathway is unique to BAFF-R among BAFF receptors [40]. Although the mechanism by which TNF-α induces CXCR5 expression in B cells has not been elucidated [28], TNF-α is known to activate the canonical NF-kB pathway [41]. Although actual signaling mechanisms on induction of CXCR5 expression in B cells by TNF-α, IL-6 and BAFF need to be further addressed, such previous findings may explain why the increased expression of CXCR5 in IgM^+^ cells by TNF-α was further enhanced by BAFF.

Since autoreactive B cells have low CXCR5 expression and reduced migratory activity towards CXCL13 [42], induction of CXCR5 expression in bone marrow by BAFF may be involved in inhibition of autoreactive B cells being out of bone marrow to the circulation. Also, since CXCR5 contributes to the formation of germinal centers after antigen presentation [21], BAFF may affect antibody production in terms of migration and localization by inducing CXCR5 expression and the effect is likely to be different in each secondary lymphoid tissue. Previous studies have shown that there is no difference between CXCR5-KO and WT mice in the migration of B cells to their regional lymph nodes, including peripheral lymph nodes such as brachial, cervical, and fascial lymph nodes, and intestinal lymph nodes [43]. Given that the CXCR5 dependency of B cell migration is greatest in the spleen, it is likely that the reduction in antigen-specific antibody production by antigen stimulation in BAFF-KO mice is most pronounced in the spleen. CXCR5 is dispensable for development of immature B cell in bone marrow as the number of circulating B cells does not decrease in CXCR5-KO mice [21], and results in this study regarding the relationship between BAFF and CXCR5 are consistent with the previous findings.

Together, this study suggests that BAFF has an important role in B cell differentiation in bone marrow by directly inducing CXCR5 expression which affect their migration to secondary lymphoid organs. Results in this study contradict the conventional view that BAFF does not play a major role in B cell differentiation in bone marrow, and rather suggest that BAFF directly induces CXCR5 expression in IgM^+^ cells in the bone marrow. The contradiction may derive from different genetic backgrounds of mice, i.e., C57BL/6 and BALB/cA backgrounds in the previous and current studies, respectively. Nonetheless, our finding may contribute to further understanding of the roles of BAFF in B cell development and establishment of humoral immunity in details.

## Declaration of competing interest

The authors declare that they have no known competing financial interests or personal relationships that could have appeared to influence the work reported in this paper.

## Data Availability

Data will be made available on request.
