# Supplementary material for: BAFF induces CXCR5 expression during B cell differentiation in bone marrow
Source: Biochem Biophys Rep. 2023 Mar 7;34:101451. doi: 10.1016/j.bbrep.2023.101451 (PMC10011739; doi:10.1016/j.bbrep.2023.101451)
Supplement: Multimedia component 1 [file mmc1.docx]

**Table 1. List of primers for Quantitative RT-PCR**

| **Gene** | **Forward** | **Reverse** |
| --- | --- | --- |
| *CD19* | 5'-ATTGCAAGGTCAGTGTGGC-3' | 5'-GAGGGAGGCGTCACTTTGAA-3' |
| *Igμ* | 5'-AAAGGATGGGAAGCTCGTGG-3' | 5'-GTCAGGTTAGCGGACTTGCT-3' |
| *Bcl-2* | 5'-TCTTTGAGTTCGGTGGGGTC-3' | 5'-ATATAGTTCCACAAAGGCATCCCAG-3' |
| *IL-7R* | 5'-GCAAGGGGTGAAAGCAACTG-3' | 5'-ACAGGATCCCATCCTCCTTGA-3' |
| *CXCR5* | 5'-TGGCCTTCTACAGTAACAGCA-3' | 5'-GCATGAATACCGCCTTAAAGGAC-3' |
| *GAPDH* | 5'-CGACTTCAACAGCAACTCCCACTCTTCC-3' | 5'-TGGGTGGTCCAGGGTTTCTTACTCCTT-3' |
